# Analyses of volatiles produced by the African fruit fly species complex (Diptera, Tephritidae)

**DOI:** 10.3897/zookeys.540.9630

**Published:** 2015-11-26

**Authors:** Radka Břízová, Lucie Vaníčková, Mária Faťarová, Sunday Ekesi, Michal Hoskovec, Blanka Kalinová

**Affiliations:** 1 Institute of Organic Chemistry and Biochemistry ASCR, v.v.i., Flemingovo nám. 2, CZ-166 10 Prague 6, Czech Republic; 2 Institute of Chemical Technology in Prague, Technická 5, CZ-166 28 Prague 6, Czech Republic; 3 Instituto de Química e Biotecnologia, Universidade Federal de Alagoas, Av. Lourival de Melo Mota, s/n, Tabuleiro, CEP 57072-970, Maceió, AL, Brazil; 4 Charles University, Albertov 6, CZ-128 43 Prague 2, Czech Republic; 5 International Centre of Insect Physiology and Ecology, PO Box 30772-00100 GPO, Nairobi, Kenya; 6 Czech University of Life Sciences, Kamýcká 129, CZ-165 21 Prague 6, Czech Republic

**Keywords:** *Ceratitis* FAR complex, chemotaxonomy, male and female-borne volatiles, GC×GC-TOFMS, GC-EAD

## Abstract

*Ceratitis fasciventris*, *Ceratitis anonae* and *Ceratitis rosa* are polyphagous agricultural pests originating from the African continent. The taxonomy of this group (the so-called *Ceratitis* FAR complex) is unclear. To clarify the taxonomic relationships, male and female-produced volatiles presumably involved in pre-mating communication were studied using comprehensive two-dimensional gas chromatography with time-of-flight mass spectrometry (GC×GC-TOFMS) followed by multivariate analysis, and gas chromatography combined with electroantennographic detection (GC-EAD). GC×GC-TOFMS analyses revealed sex specific differences in produced volatiles. Male volatiles are complex mixtures that differ both qualitatively and quantitatively but share some common compounds. GC-EAD analyses of male volatiles revealed that the antennal sensitivities of females significantly differ in the studied species. No female volatiles elicited antennal responses in males. The results show clear species-specific differences in volatile production and provide complementary information for the distinct delimitation of the putative species by chemotaxonomic markers.

## Introduction

The fruit fly family Tephritidae (Diptera) consists of four major genera, *Ceratitis*, *Bactrocera*, *Anastrepha* and *Rhagoletis*, which are considered important insect pests worldwide (Aluja and Norrbom 2001). The genus *Ceratitis* is the most studied, with *Ceratitis capitata* (Wiedemann) as species most frequently monitored and used as the model pest organism due to its global distribution. The Afro-tropical group of fruit flies *Ceratitis fasciventris* (Bezzi), *Ceratitis anonae* Graham and *Ceratitis rosa* Karsch (the so-called FAR complex) is widespread in a number of African countries: *Ceratitis fasciventris* and *Ceratitis anonae* occur sympatrically in both East and West Africa while *Ceratitis rosa* is more restricted to southern and eastern Africa where its distribution partially overlaps with that of *Ceratitis fasciventris* but not with *Ceratitis anonae* (Barr and McPheron 2006, Copeland et al. 2006). *Ceratitis rosa* is now feared to be a global threat due to its tolerance to lower temperatures (Duyck and Quilici 2002). It may expand not only within Africa, but also across Europe, Asia, Australia and the North and South American continents (De Meyer et al. 2008).

Females of the FAR complex species cause extensive damage on commercially produced fruits from 24 plant families (De Meyer et al. 2002, Copeland et al. 2006), by puncturing the fruits during oviposition and by the feeding larvae inside the fruit that generally result in premature fruit abortion. Reproduction behavior in genus *Ceratitis* is initiated by males that aggregate in leks on vegetation to lure females by releasing long-range pheromones. Females attracted by male pheromone visit leks and choose males to mate based on complex visual, acoustical and chemical stimuli (Aluja and Norrbom 2001). Chemical communication, that involves both long-range pheromones and close-range cuticular hydrocarbons, is integral part of the fruit fly courtship. Nevertheless, there are no records available on the composition of the long-range volatiles released by the FAR complex species.

Despite its economic importance, the taxonomy of this group is not clear and taxonomical classification is not easy (De Meyer and Freidberg 2006). It is particularly important to resolve invasive agricultural pest species, because inadequate morphological/molecular characterisation of the species might have serious economic consequences, resulting in inept ecological models and/or pest control strategies (Virgilio et al. 2013, Vaníčková et al. 2014).

The need to develop a precise pest-detection technique, diagnostic tools and management strategies for these pest species initiated large scale morphological and genetic studies, the investigation of their evolutionary relationships as well as the characterisation of the variation of cuticular hydrocarbon profiles within and between the species (De Meyer 2001, De Meyer and Freidberg 2006, Virgilio et al. 2012, Delatte et al. 2013, Virgilio et al. 2013, Vaníčková et al. 2014a,b, Vaníčková et al. 2015). The species of the FAR complex can only be identified based on specific small differences between the morphological characters of adult male leg patterns (larvae, pupae and females, are even more difficult to distinguish) (De Meyer and Freidberg 2006). While it is possible to identify the females of *Ceratitis anonae*, the females of *Ceratitis rosa* and *Ceratitis fasciventris* are almost indistinguishable from each other and show only very subtle differences in their scutellar colour patterns. The absence of clear diagnostic morphological features to identify individual species emphasises the need for unambiguous identification applying molecular and/or chemical tools (Baliraine et al. 2004, Vaníčková 2012, Virgilio et al. 2013, Vaníčková et al. 2014a,b). Molecular approaches for species recognition were developed in the past (Virgilio et al. 2008, Barr and Wiegmann 2009, Delatte et al. 2013). Recently, Virgilio and co-workers have provided clear data on the specification of five different morphotypes using a comparison of allelic variations at 16 microsatellite loci (Virgilio et al. 2013). Nevertheless, the use of microsatellite loci for cryptic species identification is rather laborious and expensive. Recent studies on the cuticular hydrocarbon profiles extracted from the body surface of males and females of *Ceratitis fasciventris*, *Ceratitis anonae*, *Ceratitis rosa* and *Ceratitis capitata* have supported the existence of more than three genotypes in the FAR complex (Vaníčková 2012, Vaníčková et al. 2014a,b, Vaníčková et al. 2015). The authors pinpointed several chemotaxonomic markers whose presence/absence can be used for the identification of the putative species from the FAR complex.

To understand the detailed taxonomical relationships within the FAR complex and to support the evidence on cryptic speciation presented in the aforementioned studies, we aimed to analyse the chemical composition of the volatiles emitted by males and females. The communication signals are highly species-specific and are extremely important in the reproduction isolation of different species. Therefore we assume that the specific volatiles, examined in the present study, together with cuticular hydrocarbons could serve as an effective diagnostic tool.

## Methods

### Insects

The laboratory-reared populations of *Ceratitis fasciventris*, *Ceratitis anonae* and *Ceratitis rosa* R2 type were obtained from the International Centre of Insect Physiology and Ecology (ICIPE, Nairobi, Kenya). The pupae were kept under identical laboratory conditions at the Institute of Organic Chemistry and Biochemistry (IOCB, Prague, Czech Republic). Adult flies were fed on an artificial diet consisting of sugarcane:yeast (3:1) and mineral water and were kept at a relative humidity of 60%, at 25 °C, and a 12L:12D photoperiod.

### The collection of volatiles

Male and female-borne volatiles of all three species were trapped by the standard dynamic headspace procedures. A group of five virgin 20-day-old male and/or female flies of each species was placed into round-bottom flasks (250 mL) adapted for volatile collection (Verkon, Praha, Czech Republic). Air was sucked by a pump (Pocket Pump 210 Series, SKC Inc., PA, USA) at 100 mL min^-1^ from a flask through a glass pipette-shaped filter with a sieve located at its thinner end. The filter was filled with a layer of silanised cotton (Applied Science Laboratories, Inc. Bedford, Massachusetts, USA), followed by a SuperQ® (copolymer of ethylvinylbenzene and divinylbenzene, Alltech ARS Inc., Gainesville, Florida, USA) adsorbent layer (*m* = 30 mg), and finished with another layer of glass wool and Teflon ring. The adsorbed volatiles were subsequently rinsed from the filter by 500 µL of freshly distilled HPLC quality *n*-hexane (Lachner, Neratovice, Czech Republic). The extracts were stored in the freezer until chemical analyses.

### GC×GC-TOFMS analysis

The male and female-borne headspace volatiles were identified using a LECO Pegasus 4D instrument (LECO Corp., St. Joseph, Michigan, USA). The first dimension column was a weak-polar DB-5 (J & W Scientific, Folsom, California; 30 m × 250 µm i.d. × 0.25 µm film), and the second dimension column was polar BPX-50 (SGE, Austin, Texas; 2 m × 100 µm i.d. × 0.1 µm film). 1 µL of the sample was injected in splitless mode into a constant flow of helium (1 mL min^-1^), which was used as carrier gas. Injector temperature was 220 °C; temperature in the first dimension was held for 2 min at 40 °C followed by an increase of 5 °C min^-1^ to the target temperature of 270 °C which was held for 10 min. In the second dimension the temperature program was 10 °C higher. The modulation period was 4 s, the hold pulse time was 0.6 s and the cold pulse time between the stages was set to 1.4 s. The modulation temperature offset relative to the GC oven temperature was 30 °C. The temperature of the transfer line connecting the secondary column to the TOFMS detector source was operated at 280 °C. The source temperature was 250 °C with a filament bias voltage of −70 V. The data acquisition rate was 100 scans s^-1^, with a mass range of 29–400 amu and a detector voltage of 1 650 V. The first to be analysed under the given conditions was a mixture of *n*-alkanes C_8_-C_22_ (1×10^-3^ µg µL^-1^, Sigma-Aldrich), followed by the pheromone samples. LECO ChromaTOF^TM^ is equipped with a retention index (*RI*) calculation function. The identification of analytes was based on a comparison of their mass spectra fragmentation patterns obtained by electron impact ionisation, two-dimensional retention times and retention indices with the standards available and/or previously published data. Not all the authentic standards were available though. In such cases, the identifications were carried out using the reference spectra in the NIST library, the Wiley/NBS Registry of Mass Spectral Data and published *RIs* as well as available literature (Jang et al. 1989, Light et al. 1999, Adams 2007, Vaníčková 2012, Vaníčková et al. 2012, Faťarová 2013). With regards to quantification, raw-area percentages obtained by GC were used to report the relative ratios of active compounds in the pheromone blend.

### Chemicals

The following synthetic standards were purchased from Sigma-Aldrich and tested in the concentration 5×10^-3^ µg µL^-1^: methyl (*E*)-hex-3-enoate, methyl (*E*)-hex-2-enoate, 6-methylhept-5-en-2-one, ethyl hexanoate, ethyl (*E*)-hex-3-enoate, methyl (*E*)-oct-2-enoate, geranyl acetone, (*E,E*)-α-farnesene, methyl (2*E*,6*E*)-farnesoate, linalool, (*E*)-non-2-enal, (*Z*)-non-3-enol, and (*Z*)-non-2-enol. (*Z*)-Non-3-enal and (*Z*)-non-2-enal were prepared in our laboratory from the corresponding alcohols (Hazzard 1973). The purchased and synthesised chemicals were of analytical grade purity.

### GC-EAD analyses

1–3 µL of headspace pheromone samples were injected splitless into a HP 5890 A chromatograph (Hewlet Packard, Palo Alto, CA, USA) with a Rxi-5Sil MS column (Restek, Bellefonte, PA; 30 m × 0.25 µm i.d. × 0.25 µm film). The end of the GC column was split into two arms by a Graphpack 3D/2 four-arm splitter (Gertsel Inc., Baltimore, MD, USA), directing the eluate to two detectors working simultaneously – a flame ionisation detector (FID) and an antenna (EAD). Volatile compounds were separated in a continuous helium stream (1 mL min^-1^). The parameters of the GC oven were similar to the temperature program applied at GC×GC-TOFMS and were as follows: the injector temperature was set to 200 °C and the FID filament temperature was 260 °C. The GC column was operated at a temperature program starting at 40 °C for 2 min, followed by a 10 °C min^-1^ increase until the temperature reached 270 °C, which was held for 10 min. In order to correlate GC×GC-TOFMS and GC-FID/EAD data, *RI****_EAD_*** were calculated using a standard mixture of *n*-alkanes C_8_-C_22_ (1×10^-3^µg µL^-1^) injected and analysed under the same conditions as the pheromone samples in both GC×GC-TOFMS and GC-FID/EAD systems.

The fruit fly antennal detector (EAD) was prepared by cutting off the head of a narcotised fly (virgin, 20 days old) and fixing it between two Ag/AgCl glass microelectrodes containing Ringer’s solution. The reference electrode was inserted into the head capsule and the recording one was positioned to make a contact with the sensory epithelium on the last antennomere surface. The antennal preparation was then placed in a continual air stream (1 L min^-1^) blowing from a glass tube (8 mm in diameter), in which the split GC eluate was directed. The electrical signal generated by the antennal preparation was led to a high impedance pre-amplifier (10^14^ Ω; 10× amplification, SYNTECH Equipment and Research, Kirchzarten, Germany) and fed to a PC. The data were evaluated using Syntech GC-EAD software, where signals from FID and EAD were displayed and analyzed simultaneously. Not all FID peaks elicited EAD responses. When some FID peaks were associated with EAD activity in at least 3 independent GC-EAD experiments, the compound was classified as biologically active.

To determine the antennal specificities of the three species studied, subsequent GC-EAD analyses were performed on *Ceratitis fasciventris*, *Ceratitis anonae* and *Ceratitis rosa* with equal doses of synthetic standards (10 ng). From these experiments, FID/EAD ratios were calculated from FID and EAD peak areas and compared for different compounds and species.

### Statistics

The data obtained from the chemical analysis (*N* = 7) of male emanations for each species were statistically evaluated. For the statistical analyses, the peak areas of the 22 common compounds identified in the volatile mixtures released by 20 day-old males of all three studied species of the FAR complex were used. For further analysis, only the 12 antennally active compounds were used. The differences in the chemical composition of the samples from all of the three species were analysed by principal component analysis (PCA). Prior to the PCA analysis, the peak areas were subjected to logarithmic transformation, scaling was focused on inter-species correlation, each species score was divided by its standard deviation and the data were centered by species. In the PCA analyses, samples with similar chemical profiles cluster together and segregate from those that are different. PCA was employed for unimodal data while correspondence analyses (CA) were used for linear data. The multivariate data analysis software CANOCO 4.5 (Biometris, Plant Research International, Wageningen UR, The Netherlands) was used for both the PCA and CA.

## Results

### Chemical analyses of headspace extracts of the FAR complex

**Figure 1.**
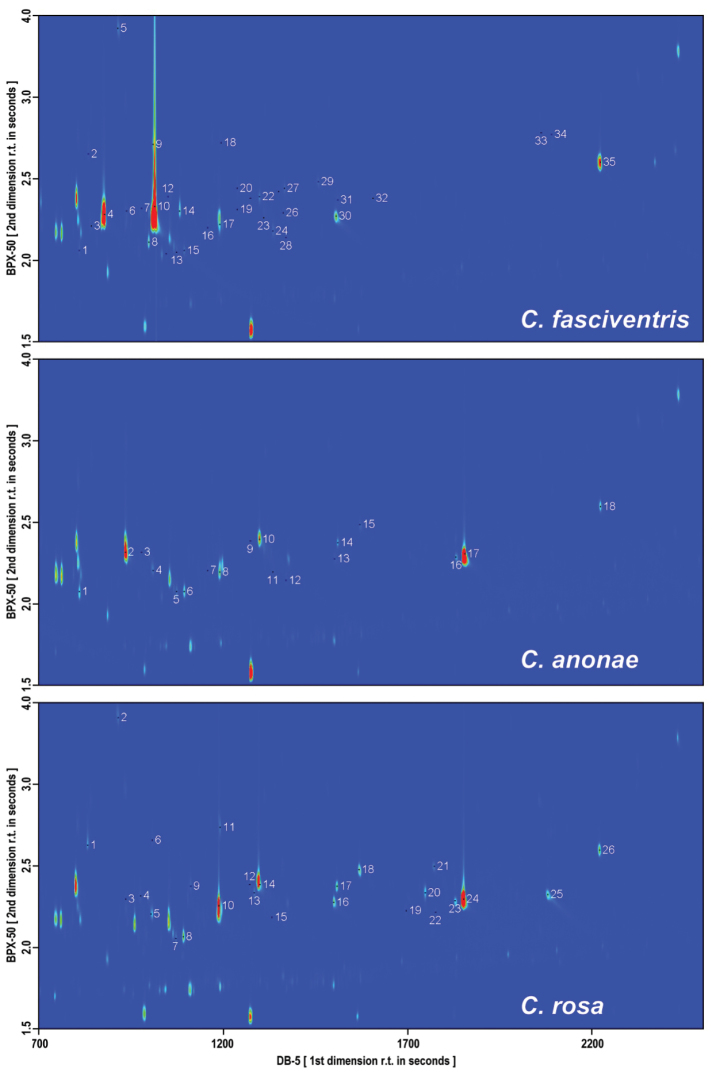
GC×GC-TOFMS chromatograms (TIC mode) of the male (*N* = 5) volatiles of *Ceratitis fasciventris*, *Ceratitis anonae* and *Ceratitis rosa*. Each spot represents one compound; the identified compounds are numbered in each chromatogram, with the numbering corresponding to the respective Table 1 of compounds. The intensity of each spot is colour-coded (blue - 0, red - maximum).

Fruit fly male-borne volatiles of *Ceratitis fasciventris*, *Ceratitis anonae*, *Ceratitis rosa* R2 type were highly complex, qualitatively and quantitatively diverse mixtures (Figure 1). GC×GC-TOFMS analysis identified 35 compounds in *Ceratitis fasciventris*, 18 compounds in *Ceratitis anonae* and 26 compounds in *Ceratitis rosa* R2 type (Suppl. materials 1–3: Table 1–3). The mean of the total chromatographic peak areas of the identified male volatiles of *Ceratitis fasciventris*, *Ceratitis anonae* and *Ceratitis rosa* R2 type reached 2.27×10^8^, 6.35×10^7^, and 4.06×10^7^, respectively. The volatile blends contained diverse chemical structures, including alcohols, aldehydes, terpenes, and esters. Out of all identified male-specific pheromone compounds, 11 were found in all studied species in varying abundance and 22 compounds were common for at least 2 species. In *Ceratitis fasciventris* the major component (≥ 8%) was ethyl (*E*)-3-hexenoate (71.5%) together with methyl (*E*)-hex-3-enoate (13.6%) and minor components (1–8%) were methyl (2*E*,6*E*)-farnesoate (4.9%), nonan-2-ol (1.6%), γ-valerolactone (1.1%), and ethyl (*E*)-hex-2-enoate (1%) (Suppl. material 1: Table 1). In *Ceratitis anonae*, the major compounds were (*E*, *E*)-α-farnesene (67%) and methyl (*E*)-hex-2-enoate (14.5%). The minor components identified comprised (*E*)-non-2-enal (6.9%), methyl (2*E*,6*E*)-farnesoate (1.9%), linalool (1.7%), (*E*)-β-ocimene (1.6%), heptan-2-ol (1.4%), (*Z*, *E*)-α-farnesene (1.1%), and octen-3-ol acetate (1%) (Suppl. material 2: Table 2). In *Ceratitis rosa*, the major components were linalool (47.8%) and (*E*)-non-2-enal (15.9%). Its minor compounds were e.g. (*E*, *E*)-α-farnesene (6.4%), (*E*)-β-ocimene (5.2%) and 6-methylhept-5-en-2-one (5%) (Suppl. material 3: Table 3). The remaining compounds identified in the male emanation of the three species studied were present in trace amounts (Suppl. materials 1–3: Table 1–3). Qualitative analysis proved that male pheromone components were not present in female volatile emanations. No female specific volatiles were detected by GC×GC-TOFMS or by GC-EAD qualitative analysis.

**Table 1.** Male-borne volatiles and their relative percentage (Area±SD) of antennaly active compounds found in the emanations of *Ceratitis fasciventris*, *Ceratitis anonae* and *Ceratitis rosa* (100% is represented by the total area of all antennaly active compounds in each respective species).

No	Compound	*RI*	*RI_EAD_*	*Ceratitis fasciventris*	*Ceratitis anonae*	*Ceratitis rosa*
1	Methyl (*E*)-hex-3-enoate	932	937	14.54 ± 2.17	-	-
2	Methyl (*E*)-hex-2-enoate	968	966	0.17 ± 0.02	14.08 ± 3.99	0.36 ± 0.45
3	6-Methylhept-5-en-2-one	988	989	0.03 ± 0.01	0.07 ± 0.08	6.59 ± 4.77
4	Ethyl hexanoate	997	999	0.93 ± 0.06	-	-
5	Ethyl (*E*)-hex-3-enoate	1003	1006	76.48 ± 4.38	-	-
6	Ethyl (*E*)-hex-2-enoate	1045	1045	1.12 ± 0.28	-	-
7	Linalool	1104	1104	0.55 ± 0.04	1.98 ± 0.57	62.44 ± 12.41
8	Methyl (*Z*)-oct-3-enoate	1131	1131	0.10 ± 0.02	-	-
9	(*E*)-Non-2-enal	1167	1163	0.83 ± 0.39	6.81 ± 1.56	20.82 ± 18.70
10	Geranyl acetone	1456	1459	-	-	0.62 ± 0.01
11	(*E*, *E*)-α-Farnesene	1507	1507	-	74.81 ± 20.93	8.31 ± 0.86
12	Methyl (2*E*,6*E*)-farnesoate	1798	1799	5.26 ± 0.91	2.25 ± 0.82	0.86 ± 0.26

*RI* retention index identified by GC×GC-TOFMS; *RI_EAD_* retention index of antennaly active compounds identified using GC-FID/EAD.

*Ceratitis fasciventris* was found to have the highest number of species-specific compounds that were not found in other two species (Table 1, Suppl. material 1: Table 1). Pheromone specificity in this fruit fly was based on the presence of 17 compounds, e.g. saturated and unsaturated esters of isomers of hexenoic acid, specifically methyl (*E*)-hex-3-enoate (*RI* = 932), ethyl hexanoate (*RI* = 997), ethyl (*E*)-hex-3-enoate (*RI* = 1003), ethyl (*E*)-hex-2-enoate (*RI* = 1045), and methyl (*Z*)-oct-3-enoate (*RI* = 1131). These esters were absent in *Ceratitis rosa* and *Ceratitis anonae* male pheromone emanations (Table 1, Suppl. materials 2 and 3: Table 2, 3). The only ester of hexenoic acid shared by all three species studied was methyl (*E*)-hex-2-enoate (*RI* = 968). Furthermore, *Ceratitis fasciventris* did not emit isomers of α or β-farnesenes when compared with the other two species (Suppl. material 1: Table 1). Interestingly, *Ceratitis anonae* had no specific compounds. *Ceratitis rosa* had six species-specific compounds: (*E*)-oct-2-enal (*RI* = 1062), (*Z*)-non-3-enol (*RI* = 1158), β-elemene (*RI* = 1406), β-caryophyllene (*RI* = 1442), geranyl acetone (*RI* = 1456), and (*Z*)-β-farnesene (*RI* = 1458).

### Electrophysiological analyses

**Figure 2.**
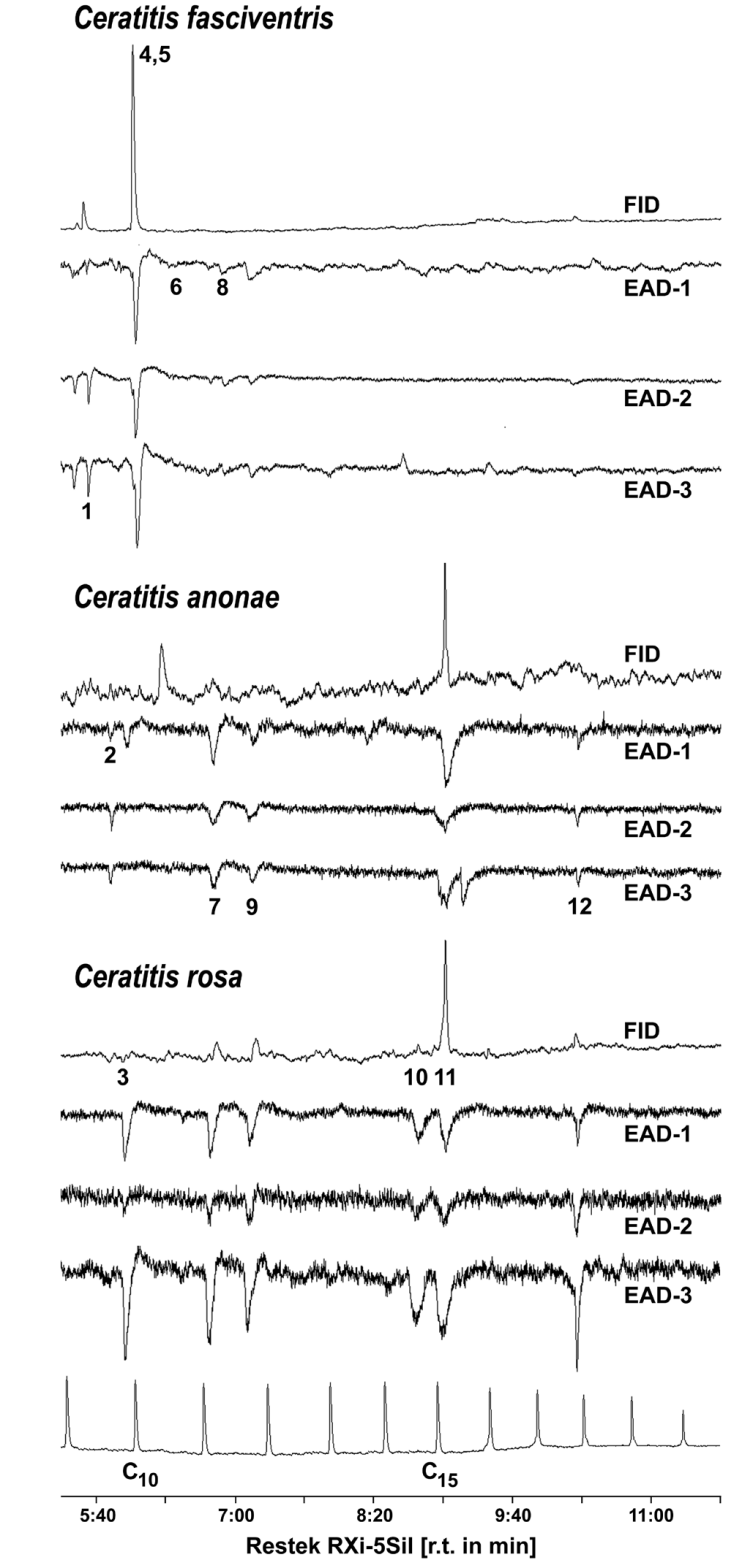
GC-FID/EAD analyses of the *Ceratitis fasciventris*, *Ceratitis anonae*, and *Ceratitis rosa* male-borne volatiles using a conspecific female antenna as an EAD detector. The numbers indicate EAD-active compounds and correspond to Table 1. The symbols EAD-1-3 denote the three independent repetitions of the GC-EAD analyses.

The biological activity of the male volatile components present in the headspace samples was examined using female and male antennae and the GC-EAD technique. The antennal depolarisation was triggered by 12 compounds in total (Table 1, Figure 2). Out of these, five components, methyl (*E*)-hex-2-enoate (*RI*_EAD_ = 966), 6-methylhept-5-en-2-one (*RI*_EAD_ = 989), linalool (*RI*_EAD_ = 1104), (*E*)-non-2-enal (*RI*_EAD_ = 1163) and methyl (2*E*,6*E*)-farnesoate (*RI*_EAD_ = 1799), elicited biological activity in all of the species studied (Table 1, Figure 2). Five EAD-active compounds were specific for *Ceratitis fasciventris*, methyl (*E*)-hex-3-enoate (*RI*_EAD_ = 937), ethyl hexanoate (*RI*_EAD_ = 999), ethyl (*E*)-hex-3-enoate (*RI*_EAD_ = 1006), ethyl (*E*)-hex-2-enoate (*RI*_EAD_ = 1045) and methyl (*Z*)-oct-3-enoate (*RI*_EAD_ = 1131), whereas *Ceratitis rosa* had one EAD-specific compound – geranyl acetone (*RI*_EAD_ = 1459). (*E*, *E*)-α-Farnesene (*RI*_EAD_ = 1507) was EAD-active compounds shared by *Ceratitis anonae* and *Ceratitis rosa*.

**Figure 3.**
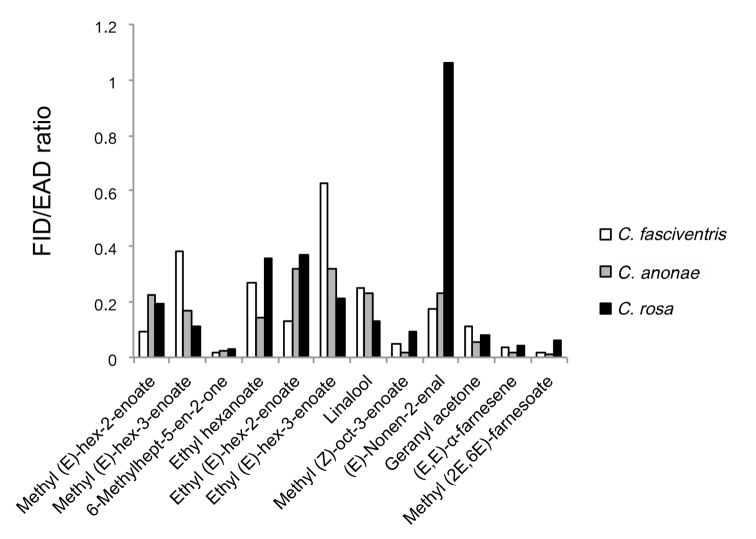
A comparison of female antennal responses of *Ceratitis fasciventris*, *Ceratitis anonae*, and *Ceratitis rosa* to standard solutions. The FID/EAD on the y-axis represents the ratio between an electroantennographic response and a conventional detector. The higher the number, the higher the response (*N* = 3).

There was no correlation between the amplitude of the EAD response and the relative abundance of the volatiles identified from the headspace male pheromone analysis. Among the three FAR complex species, the ranking of the relative EAD responses was specific for the respective species (Figure 3). The effectiveness of individual compounds is expressed as an area ratio of the respective FID and EAD responses obtained from GC-EAD analyses. The higher the FID/EAD ratio, the higher the antennal sensitivity to the respective synthetic compound (*N* = 3). In *Ceratitis fasciventris*, females respond with the highest sensitivity to ethyl (*E*)-hex-3-enoate, the most abundant compound in the *Ceratitis fasciventris* male pheromone. Methyl (*E*)-hex-3-enoate is the second most abundant compound eliciting the second highest antennal response in this species, followed by ethyl hexanoate, linalool, and (*E*)-non-2-enal. Methyl (2*E*,6*E*)-farnesoate was the least effective compounds among all the synthetic standards tested in *Ceratitis fasciventris*. Among antennaly active components of *Ceratitis anonae* pheromone, (*E*, *E*)-α-farnesene is the most abundant, but it does not yield prominent response in *Ceratitis anonae* males. Instead, the *Ceratitis anonae* responded almost equally to ethyl-(*E*)-hex-2-enoate and ethyl-(*E*)-hex-3-enoate, followed by linalool, (*E*)-non-2-enal, methyl (*E*)-hex-2-enoate, and methyl (*E*)-hex-3-enoate. The less efficient compound along with (*E,E*)-α-farnesene is methyl (2*E*,6*E*)-farnesoate. The antennae of *Ceratitis rosa* females revealed prominent response to (*E*)-non-2-enal. Other compounds like ethyl (*E*)-hex-2-enoate, ethyl hexanoate, methyl (*E*)-hex-2-enoate, methyl (*E*)-hex-3-enoate, linalool, methyl (*Z*)-oct-3-enoate, geranyl acetone, 6-methylhept-5-en-2-one, (*E,E*)-α-farnesene, and methyl (2*E*,6*E*)-farnesoate were less active.

The female antennae of the three species have distinct specificities related to a conspecific pheromone (Figure 3). Nevertheless, they can also perceive pheromone components of the other two species. Both male and female antennae respond to the male-borne volatiles (data not presented).

### PCA and CA analyses

**Figure 4.**
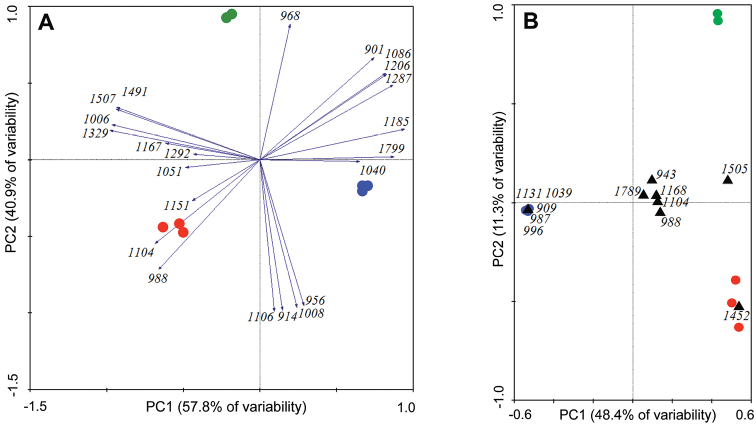
The results of statistical analyses of the male-borne volatiles produced by *Ceratitis fasciventris* (blue), *Ceratitis anonae* (green) and *Ceratitis rosa* (red). (**A**) Multivariate principal component analysis (PCA) of the 22 common compounds identified in the pheromone of the males of the FAR complex. (**B**) Multivariate correspondence analysis (CA) of the 12 antennal active compounds. The three species are clearly segregated. Each symbol on the plot represents one sample. The numbers in italics denote the retention indices (*RI*) of the species-specific compounds. For the structural identification of the compounds see the Suppl. materials 1–3: Tables 1–3.

The results of the principal component analyses are depicted in Figure 4A. The PCA shows a clear separation of the three species, indicating that the composition of the male pheromones is specific in each of the species. The two principal components (PC1 and PC2) together accounted for 97% of the total variability. The 22 common pheromone compounds are presented by the numbers in italics, which stand for the particular retention indices (Suppl. materials 1–3). The compounds specific for *Ceratitis fasciventris* male emanations were 2,5-dimethylpyrazine (*RI* = 914), γ-valerolactone (*RI* = 956), ethyl (*E*)-hex-3-enoate (*RI* = 1106), 2,3,5-trimethylpyrazine (*RI* = 1108) and (Z)-β-ocimene (*RI* = 1040). The *Ceratitis anonae* pheromone was characterised by octanal (*RI* = 1006), (E)-non-2-enal (*RI* = 1167), oct-3-enyl acetate (*RI* = 1292), methyl geranate (*RI* = 1329), (*Z,E*)-α-farnesene (*RI* = 1491), and (*E,E*)-α-farnesene (*RI* = 1507). 6-Methylhept-5-en-2-one (*RI* = 988), (*E*)-β-ocimene (*RI* = 1051), linalool (*RI* = 1104) and (*Z*)-non-2-enal (*RI* = 1151) were the compounds specific for the *Ceratitis rosa* male pheromone.

The multivariate correspondence analyses (CA) of the 12 EAD-active compounds identified by GC×GC-MS and GC-FID/EAD are depicted in Figure 4B. The results reveal species-specific EAD compounds identified by their retention indices. The FAR complex species formed three segregated groups. The CA analyses have revealed exclusive compounds for particular species. Methyl (*E*)-hex-3-enoate (*RI*_EAD_ = 932), ethyl hexanoate (*RI*_EAD_ = 996), ethyl (*E*)-hex-2-enoate (*RI*_EAD_ = 1045) and methyl (*Z*)-oct-3-enoate (*RI*_EAD_ = 1131) are characteristic for the aeration extract from *Ceratitis fasciventris*. The *Ceratitis anonae* emanation also has typical compounds: methyl (*E*)-hex-2-enoate (*RI*_EAD_ = 968), (*E*)-non-2-enal (*RI*_EAD_ = 1163), (*E*, *E*)-α-farnesene (*RI*_EAD_ = 1507), and methyl (2*E*,6*E*)-farnesoate (*RI*_EAD_ = 1799). *Ceratitis rosa* is defined by geranyl acetone (*RI*_EAD_ = 1459) and 6-methylhept-5-en-2-one (*RI*_EAD_ = 988). Terpene linalool (*RI*_EAD_ = 1104) is shared by *Ceratitis anonae* and *Ceratitis rosa*.

## Discussion

The present study provides the first identification and biological evaluation of volatiles produced by the *Ceratitis* FAR complex species. Our data show that pheromones in the study species are produced exclusively by males and are, like in other fruit fly species (Aluja and Norrbom 2001), highly complex species-specific mixtures characterized by specific qualitative and quantitative profiles of diverse chemical structures, including alcohols, aldehydes, terpenes, and esters.

The techniques applied in the present study (headspace collection of insect volatiles, GC×GC-TOFMS, GC-EAD) allowed for detail analyses and identification of the specific volatiles produced by the FAR complex species. The headspace technique using the glass filter with the adsorbent is suitable when the extract from the same aeration/sample needs to be analysed by different approaches, e.g. GC-MS, GC×GC-TOFMS, GC-EAD, and behavioral assays (Vaníčková et al. 2012, Břízová et al. 2013, Gonçalves et al. 2013, Milet-Pinheiro et al. 2015). On the other hand the SPME fiber could be also use for the qualitative analyses of the fruit fly emanation (Alfaro et al. 2011, Cruz-López et al. in press). Nevertheless due to impossible storage of the SPME fiber for further laboratory bioassays, the dynamic headspace technique was preferred.

Our GC×GC-TOFMS analysis of the *Ceratitis* FAR complex pheromones resulted in the identification of 35 compounds produced by *Ceratitis fasciventris* males, 18 compounds released by *Ceratitis anonae* males and 26 volatiles emitted by *Ceratitis rosa* R2 type males. The composition of the male sex pheromones in the three species partially overlaps (11 compounds were shared among all three species, but were present in species-specific quantities). In addition to common compounds, the three respective species released also species-specific compounds. *Ceratitis fasciventris* had the highest number of specific compounds. The pheromone specificity in this fruit fly is based on the presence of saturated and unsaturated methylated and ethylated esters of hexenoic acid, specifically methyl (*E*)-hex-3-enoate, ethyl hexanoate, ethyl (*E*)-3-hexenoate and ethyl (*E*)-2-hexenoate. These esters are absent from *Ceratitis rosa* and *Ceratitis anonae* male pheromone emanations. The only ester of hexenoic acid shared by all three studied species is methyl (*E*)-hex-2-enoate. Furthermore, the males of *Ceratitis fasciventris* do not emit isomers of α or β-farnesene. *Ceratitis anonae* has no specific compound. The male pheromone of *Ceratitis rosa* has 6 species-specific compounds: (*E*)-oct-2-enal, (*Z*)-non-3-enol, β-elemene, β-caryophyllene, geranyl acetone and (*Z*)-β-farnesene.

Among all identified compounds in the *Ceratitis* FAR complex species, only a relatively small set of 12 compounds elicited antennal responses suggesting their prominent roles in pheromone communication. Biological activity was elicited by four compounds found in emanations of all three species studied, namely methyl (*E*)-hex-3-enoate, 6-methylhept-5-en-2-one, linalool, (*E*)-non-2-enal and methyl (2*E*,6*E*)-farnesoate. These shared compounds are present in the respective species in quite different amounts. In addition to the shared antennally active compounds, (*E,E*)-α-farnesene triggered antennal responses in the pheromones of *Ceratitis anonae* and *Ceratitis rosa* and geranyl acetone was an active component of the *Ceratitis rosa* pheromone.

GC-EAD data have shown that the females of the three investigated species of the *Ceratitis* FAR complex perceive the components of conspecific pheromones, but are also able to perceive the pheromone components of the other two species. The antennal responses of individual species differ significantly and are species-specific. Both males and females can perceive the male pheromone.

Many of the *Ceratitis* FAR complex male-borne volatiles identified here [e.g. 2,5-dimethylpyrazine, 6-methyhept-5-en-2-one, ethyl (*E*)-oct-3-enoate, (*Z*)-β-ocimene, (*E*)-β-ocimene, linalool, geranyl acetone, and α-farnesenes] have been previously reported as a part of male emanations of other fruit fly pheromones (Jang et al. 1989, Flath et al. 1993, Light et al. 1999, Cossé et al. 1995, Alfaro et al. 2011, Vaníčková 2012, Vaníčková et al. 2012). Nevertheless, some of the components, e.g. methyl (2*E*,6*E*)-farnesoate and (*E*)-non-2-enal, are reported here for the first time as constituents of male emanations in the genera *Ceratitis*. Among antennaly active compounds, saturated and unsaturated ethylated and methylated esters of hexenoic acid represent volatile components of many kinds of fruits and food. Some of them were reported as constituents of the pheromones of the Mediterranean fruit fly, *Ceratitis capitata* (Jang et al. 1989, Light et al. 1999, Vaníčková 2012, Vaníčková et al. 2012), *Anastrepha ludens* and *Anastrepha obliqua* (Robacker 1988, López-Guillén et al. 2011). An ubiquitous plant compound, 6-methyhept-5-en-2-one was also determined as an antennally active component of the male sex pheromone in *Ceratitis capitata* (Vaníčková 2012, Vaníčková et al. 2012). A widely distributed plant volatile, (*E*, *E*)-α-farnesene, is an antennally and behaviourally active pheromone component in four tephritid species *Ceratitis capitata* (Jang et al. 1989, Heath et al. 1991, Vaníčková 2012, Vaníčková et al. 2012), *Anastrepha ludens*, *Anastrepha suspensa* (Rocca et al. 1992, Lu and Teal 2001) and *Anastrepha fraterculus* (Lima-Mendonça et al. 2014, Milet-Pinheiro et al. 2015). Isoprenoid geranyl acetone, aliphatic volatile (*E*)-non-2-enal and methyl (2*E*,6*E*)-farnesoate have not yet been reported in any fruit fly male sex pheromone. Geranyl acetone and (*E*)-non-2-enal are widely distributed plant volatiles that might be sequestrated from the plants to become part of the pheromone. What is very interesting is the presence of methyl (2*E*,6*E*)-farnesoate, which is a Crustacean reproductive hormone that is structurally similar to the insect juvenile hormone. In Crustacea, methyl (2*E*,6*E*)-farnesoate is responsible for enhancing reproductive maturation, maintaining juvenile morphology, and influencing male sex determination (Olmstead and LeBlanc 2007, Nagaraju and Borst 2008). In insects, methyl (2*E*,6*E*)-farnesoate is an immediate precursor of the insect juvenile hormone III (Teal et al. 2014) with the same function as in Crustacea. As a semiochemical, methyl (2*E*,6*E*)-farnesoate was reported only once in the pentatomid bug pheromone (Millar et al. 2002).

## Conclusion

Our data show that the male-borne volatile profiles of the studied species of *Ceratitis fasciventris*, *Ceratitis anonae* and *Ceratitis rosa* differ both qualitatively and quantitatively. Also antennal responses to volatile compounds are species-specific in the three species studied. Therefore, the pheromone composition as well as electroantennography may be used as specific tools for the FAR complex species delimitation. Our findings are in agreement with recent studies on the cuticular hydrocarbon profiles of *Ceratitis fasciventris*, *Ceratitis anonae*, *Ceratitis rosa* R1 and R2 type and *Ceratitis capitata*, which shows that the cuticular fingerprints are species and sex-specific (Vaníčková 2012, Vaníčková et al. 2014a,b, Vaníčková et al. 2015). Like the composition of cuticular hydrocarbons, also pheromone composition and antennal specificity suggest that the *Ceratitis* FAR complex species are taxonomically well-defined entities.

## Supplementary materials

Supplementary material 1 Table 1 Data type: Adobe PDF file Explanation note: Compounds, their relative percentage (Area±SD), and chemical characteristics identified by GC×GC-TOFMS and GC-FID/EAD in the headspace extracts of the calling males of *Ceratitis fasciventris*. This dataset is made available under the Open Database License (http://opendatacommons.org/licenses/odbl/1.0/). The Open Database License (ODbL) is a license agreement intended to allow users to freely share, modify, and use this Dataset while maintaining this same freedom for others, provided that the original source and author(s) are credited.Radka Břízová, Lucie Vaníčková, Mária Faťarová, Sunday Ekesi, Michal Hoskovec, Blanka Kalinová

Supplementary material 2 Table 2 Data type: Adobe PDF file Explanation note: Compounds, their relative percentage (Area±SD), and chemical characteristics identified by GC×GC-TOFMS and GC-FID/EAD in the headspace extracts of the calling males of *Ceratitis anonae*. This dataset is made available under the Open Database License (http://opendatacommons.org/licenses/odbl/1.0/). The Open Database License (ODbL) is a license agreement intended to allow users to freely share, modify, and use this Dataset while maintaining this same freedom for others, provided that the original source and author(s) are credited.Radka Břízová, Lucie Vaníčková, Mária Faťarová, Sunday Ekesi, Michal Hoskovec, Blanka Kalinová

Supplementary material 3 Table 3 Data type: Adobe PDF file Explanation note: Compounds, their relative percentage (Area±SD), and chemical characteristics identified by GC×GC-TOFMS and GC-FID/EAD in the headspace extracts of the calling males of *Ceratitis rosa*. This dataset is made available under the Open Database License (http://opendatacommons.org/licenses/odbl/1.0/). The Open Database License (ODbL) is a license agreement intended to allow users to freely share, modify, and use this Dataset while maintaining this same freedom for others, provided that the original source and author(s) are credited.Radka Břízová, Lucie Vaníčková, Mária Faťarová, Sunday Ekesi, Michal Hoskovec, Blanka Kalinová
