# Supplementary material for: Analyses of volatiles produced by the African fruit fly species complex (Diptera, Tephritidae)
Source: Zookeys. 2015 Nov 26;(540):385–404. doi: 10.3897/zookeys.540.9630 (PMC4714079; doi:10.3897/zookeys.540.9630)
Supplement: Supplementary material 1 — Table 1 [file zookeys-540-385-s001.pdf]

Supplementary file 1: Table 1 Compounds, their relative percentage (Area±SD), and chemical characteristics identified by GC×GC-TOFMS and GC-FID/EAD in the headspace extracts of the calling males of *Ceratitis fasciventris*.

No	Compound	*RI*	RI_{EAD}	t_R [s]	Area±SD
1	Heptan-2-ol[†]	901		806, 2.060	0.13 ± 0.01
2	2,5-Dimethylpyrazine	914		830, 2.650	0.23 ± 0.01
3	Ethyl 4-pentenoate[†]	916		834, 2.220	0.27 ± 0.00
4	Methyl (*E*)-hex-3-enoate[§,‡]	932	937	870, 2.230	13.59 ± 1.01
5	γ-Valerolactone[a]	956		910, 3.420	1.07 ± 0.33
6	Methyl (*E*)-hex-2-enoate[§,‡]	968	966	934, 2.300	0.16 ± 0.02
7	6-Methylhept-5-en-2-one[§,‡]	988	989	974, 2.330	0.03 ± 0.01
8	Ethyl hexanoate[§,‡]	997	999	990, 2.120	0.87 ± 0.05
9	Ethyl (*E*)-hex-3-enoate[§]	1003	1006	1002 2.230	71.49 ± 4.11
10	2,3,5-Trimethylpyrazine	1008		1006, 2.710	0.44 ± 0.03
11	2-Pentylbutyrate[†]	1025		1042, 2.040	0.03 ± 0.01
12	Unknown 1	1027		1046, 2.380	0.07 ± 0.01
13	(*Z*)-β-Ocimene[†]	1040		1070, 2.050	0.32 ± 0.01
14	Ethyl (E)-hex-2-enoate[§,‡]	1045	1045	1078, 2.300	1.04 ± 0.26
15	(*E*)-β-Ocimene[†]	1051		1090, 2.060	0.24 ± 0.03
16	Unknown 2	1086		1154, 2.210	0.10 ± 0.00
17	Linalool[§,‡]	1104	1104	1186, 2.220	0.51 ± 0.04
18	Unknown 3	1106		1190, 2.720	0.08 ± 0.01
19	Methyl (*Z*)-oct-3-enoate[§]	1131	1131	1234, 2.310	0.10 ± 0.02
20	Ethyl 3-hydroxyhexanoate	1133		1238, 2.430	0.13 ± 0.05
21	(*Z*)-Non-2-enal[‡]	1151		1270, 2.380	0.01 ± 0.00
22	(*E*)-Non-2-enal[§,‡]	1167	1163	1298, 2.390	0.77 ± 0.36
23	(*E*)-Non-2-enol	1172		1306, 2.310	0.16 ± 0.05
24	Unknown 4	1185		1330, 2.180	0.37 ± 0.04
25	(*Z*)-Non-2-en-4-yn-1-ol	1195		1346, 2.420	0.08 ± 0.01
26	Ethyl (*Z*)-oct-3-enoate[†]	1201		1358, 2.290	0.12 ± 0.01
27	4-Ethyl-hex-2-ynal	1204		1362, 2.440	0.18 ± 0.03
28	Unknown 5	1206		1366, 2.140	0.11 ± 0.06
29	Unknown 6	1260		1454, 2.480	0.25 ± 0.18
30	Nonan-2-ol	1287		1498, 2.290	1.63 ± 0.64
31	Octen-3-ol acetate	1292		1506, 2.380	0.19 ± 0.05
32	Unknown 7	1352		1602, 2.380	0.06 ± 0.02
33	Unknown 8	1722		2054, 2.790	0.04 ± 0.01
34	Unknown 9	1735		2082, 2.780	0.11 ± 0.02
35	Methyl (2*E*,6*E*)-farnesoate[§,‡]	1798	1799	2218, 2.600	4.92 ± 0.85

RI retention index identified by GC×GC-TOFMS; RI_{EAD} retention index of antennaly active compounds identified using GC-FID/EAD, t_R retention time on first (DB-5) and second (BPX-50) column; [†]compounds identified using published mass spectral data; [‡]compounds tested using commercial or laboratory prepared standards; [§]antennaly active compounds.
