# Supplementary material for: Analyses of volatiles produced by the African fruit fly species complex (Diptera, Tephritidae)
Source: Zookeys. 2015 Nov 26;(540):385–404. doi: 10.3897/zookeys.540.9630 (PMC4714079; doi:10.3897/zookeys.540.9630)
Supplement: Supplementary material 2 — Table 2 [file zookeys-540-385-s002.pdf]

Supplementary file 2: Table 2 Compounds, their relative percentage (Area±SD), and chemical characteristics identified by GC×GC-TOFMS and GC-FID/EAD in the headspace extracts of the calling males of *Ceratitis anonae*.

No	Compound	*RI*	RI_{EAD}	t_R [s]	Area±SD
1	Heptan-2-ol[†]	901		806, 2.060	1.44 ± 0.49
2	Methyl (*E*)-hex-2-enoate[§]	968	966	930, 2.310	14.46 ± 3.84
3	6-Methylhept-5-en-2-one[§,‡]	988		974, 2.330	0.05 ± 0.07
4	Octanal	1006		1006, 2.200	0.65 ± 0.26
5	(*Z*)-β-Ocimene[†]	1040		1070, 2.070	0.58 ± 0.26
6	(*E*)-β-Ocimene[†]	1051		1090, 2.070	1.58 ± 0.59
7	Unknown 2	1086		1154, 2.200	0.17 ± 0.10
8	Linalool[§,‡]	1104	1104	1186, 2.180	1.77 ± 0.55
9	(*Z*)-Non-2-enal[‡]	1151		1270, 2.380	0.09 ± 0.03
10	(*E*)-Non-2-enal[§,‡]	1167	1163	1298, 2.380	6.90 ± 1.50
11	Unknown 4	1185		1330, 2.190	0.16 ± 0.04
12	Unknown 5	1206		1366, 2.140	0.18 ± 0.06
13	Nonan-2-ol	1287		1498, 2.270	0.37 ± 0.08
14	Octen-3-ol acetate	1292		1506, 2.370	1.05 ± 0.04
15	Methyl geranate[†]	1329		1566, 2.480	0.43 ± 0.09
16	(*Z,E*)-α-Farnesene[a]	1491		1826, 2.280	1.12 ± 0.27
17	(*E,E*)-α-Farnesene[§,†]	1507		1850, 2.300	67.01 ± 20.11
18	Methyl (2*E*,6*E*)-farnesoate[§,‡]	1798	1799	2218, 2.590	1.97 ± 0.79

RI retention index identified by GC×GC-TOFMS; RI_{EAD} retention index of antennaly active compounds identified using GC-FID/EAD, t_R retention time on first (DB-5) and second (BPX-50) column; [†]compounds identified using published mass spectral data; [‡]compounds tested using commercial or laboratory prepared standards; [§]antennaly active compounds.
