# Supplementary material for: Analyses of volatiles produced by the African fruit fly species complex (Diptera, Tephritidae)
Source: Zookeys. 2015 Nov 26;(540):385–404. doi: 10.3897/zookeys.540.9630 (PMC4714079; doi:10.3897/zookeys.540.9630)
Supplement: Supplementary material 3 — Table 3 [file zookeys-540-385-s003.pdf]

Supplementary file 3: Table 3 Compounds, their relative percentage (Area±SD), and chemical characteristics identified by GC×GC-TOFMS and GC-FID/EAD in the headspace extracts of the calling males of *Ceratitis rosa*.

No	Compound	*RI*	RI_{EAD}	t_R [s]	Area±SD
1	2,5-Dimethylpyrazine	914		830, 2.620	4.27 ± 0.25
2	γ-Valerolactone†	956		910, 3.440	1.97 ± 0.76
3	Methyl (*E*)-hex-2-enoate§,‡	968	966	934, 2.290	0.28 ± 0.04
4	6-Methylhept-5-en-2-on§,‡	988	989	978, 2.130	5.04 ± 0.65
5	Octanal	1005		1002, 2.210	2.13 ± 0.85
6	2,3,5-Trimethylpyrazine	1008		1006, 2.650	2.28 ± 0.79
7	(*Z*)-β-Ocimene†	1040		1070, 2.050	0.83 ± 0.03
8	(*E*)-β-Ocimene†	1053		1090, 2.060	5.24 ± 0.78
9	(*E*)-Oct-2-enal†	1062		1106, 2.380	0.79 ± 0.08
10	Linalool§,‡	1104	1104	1186, 2.150	47.77 ± 2.49
11	Unknown 3	1106		1190, 2.750	2.28 ± 0.79
12	(*Z*)-Non-2-enal‡	1151		1270, 2,380	0.17 ± 0.01
13	(*Z*)-Non-3-enol‡	1158		1282, 2.330	1.29 ± 0.19
14	(*E*)-Non-2-enal§,‡	1167	1163	1302, 2.370	15.92 ± 4.31
15	Unknown 4	1185		1330, 2.180	0.05 ± 0.02
16	Nonan-2-ol	1287		1498, 2.270	1.34 ± 0.12
17	Octen-3-ol acetate	1292		1506, 2.370	3.88 ± 0.34
18	Methyl geranate†	1329		1574, 2.450	1.06 ± 0.46
19	β-Elemene†	1406		1690, 2.230	0.03 ± 0.00
20	β-Caryophyllene†	1442		1742, 2.340	0.22 ± 0.04
21	Geranyl acetone§,‡	1456	1459	1770, 2.480	0.48 ± 0.00
22	(*Z*)-β-Farnesene†	1458		1774, 2.210	0.05 ± 0.03
23	(*Z,E*)-α-Farnesene†	1494		1830, 2.270	0.22 ± 0.01
24	(*E,E*)-α-Farnesene§,‡	1507	1507	1850, 2.290	6.36 ± 0.54
25	Tridecan-1-ol	1735		2078, 2.320	0.43 ± 0.34
26	Methyl (2*E*,6*E*)-farnesoate§,‡	1798	1799	2214, 2.600	0.66 ± 0.20

RI retention index identified by GC×GC-TOFMS; RI_{EAD} retention index of antennaly active compounds identified using GC-FID/EAD, t_R retention time on first (DB-5) and second (BPX-50) column; †compounds identified using published mass spectral data; ‡compounds tested using commercial or laboratory prepared standards; §antennaly active compounds.
